# Effect of *tt*-farnesol and myricetin on in vitro biofilm formed by *Streptococcus mutans* and *Candida albicans*

**DOI:** 10.1186/s12906-018-2132-x

**Published:** 2018-02-14

**Authors:** Guilherme Roncari Rocha, Elkin Jahir Florez Salamanca, Ana Letícia de Barros, Carmélia Isabel Vitorino Lobo, Marlise Inêz Klein

**Affiliations:** Department of Dental Materials and Prosthodontics, São Paulo State University (Unesp), School of Dentistry, Araraquara, Rua Humaitá, 1680, Araraquara, Sao Paulo 14801-903 Brazil

**Keywords:** Topical treatment, *tt*-farnesol, Myricetin, Cariogenic biofilm, *Streptococcus Mutans*, *Candida albicans*

## Abstract

**Background:**

Dental caries is considered a multifactorial disease, in which microorganisms play an important role. The diet is decisive in the biofilm formation because it provides the necessary resources for cellular growth and exopolysaccharides synthesis. Exopolysaccharides are the main components of the extracellular matrix (ECM). The ECM provides a 3D structure, support for the microorganisms and form diffusion-limited environments (acidic niches) that cause demineralization of the dental enamel. *Streptococcus mutans* is the main producer of exopolysaccharides. *Candida albicans* is detected together with *S. mutans* in biofilms associated with severe caries lesions. Thus, this study aimed to determine the effect of *tt*-farnesol and myricetin topical treatments on cariogenic biofilms formed by *Streptococcus mutans* and *Candida albicans*.

**Methods:**

In vitro dual-species biofilms were grown on saliva-coated hydroxyapatite discs, using tryptone-yeast extract broth with 1% sucrose (37 °C, 5% CO_2_). Twice-daily topical treatments were performed with: vehicle (ethanol 15%, negative control), 2 mM myricetin, 4 mM *tt-*farnesol, myricetin + *tt-*farnesol, myricetin + *tt-*farnesol + fluoride (250 ppm), fluoride, and chlorhexidine digluconate (0.12%; positive control). After 67 h, biofilms were evaluated to determine biofilm biomass, microbial population, and water-soluble and -insoluble exopolysaccharides in the ECM.

****Results**:**

Only the positive control yielded a reduced quantity of biomass and microbial population, while *tt-*farnesol treatment was the least efficient in reducing *C. albicans* population. The combination therapy myricetin + farnesol + fluoride significantly reduced water-soluble exopolysaccharides in the ECM (vs. negative control; *p* < 0.05; ANOVA one-way, followed by Tukey’s test), similarly to the positive control.

****Conclusions**:**

Therefore, the combination therapy negatively influenced an important virulence trait of cariogenic biofilms. However, the concentrations of both myricetin and *tt-*farnesol should be increased to produce a more pronounced effect to control these biofilms.

## Background

Dental caries represents one of the most prevalent human diseases worldwide and is becoming a worrying public health problem [1–3]. It is a chronic disease characterized by localized demineralization of dental structures, caused by microbial metabolic products, specifically organic acids coming from dental plaque (oral biofilm). The establishment of a cariogenic biofilm occurs due to an imbalance of complex interactions between oral microorganisms, host and dietary factors [4, 5]. Biofilms are defined as highly dynamic microbial communities immersed in an extracellular matrix (ECM). The ECM provides a three-dimensional (3D) structure and increases acidic niches that restrict the access of buffering saliva [6–8]. Preventing such biofilm formation is paramount to prevent dental caries occurrence.

In case of extremely destructive caries lesion with rapid progression (i.e., severe early childhood caries or S-ECC), the microorganisms *Streptococcus mutans* and *Candida albicans* are detected, concomitantly with a high intake of dietary sugars [9, 10]. When sucrose is present the adhesion between these two organisms is enhanced [11, 12]. Moreover, the symbiotic interactions between *S. mutans* and *C. albicans* increased acid production and extracellular glucan formation, enabling the assembly of a dense and abundant matrix rich in exopolysaccharides (EPS) [11, 13]. The EPS will behave as a bridge between fungal and bacterial cells [12].

*S. mutans* is widely recognized as an etiological factor of dental caries. This species is acidogenic and aciduric [14, 15], and encodes exoenzymes glucosyltransferases (Gtfs) that synthesize EPS (when sucrose is available) [16]. *S. mutans* is the main producer of ECM in dental biofilms [16]. The Gtfs are also components of saliva-pellicle and foment adhesion and accumulation of *S. mutans* and other microorganisms [17], including *C. albicans* that provides an abundance of binding sites for Gtfs derived from *S. mutans* [11].

*C. albicans* is the most commonly detected fungal organism on human mucosal surfaces and co-adheres with other commensal species, helping in biofilm formation [18, 19], when properly sugar resources are available in the diet [20]. This fungus has an extraordinary acid production and tolerance capability, besides aspartyl protease secretion [21, 22]. These exoenzymes are capable of degrading dentinal collagen under acidic conditions, increasing the cariogenic potential of biofilms [21, 22].

Commonly therapies have a microorganism as a target (as an individual causative agent); however, treatments for infectious diseases should consider a polymicrobial cause, where the interactions between microorganism can increase pathogenicity [23], and the 3D structure of ECM that protects microorganisms from antimicrobial agents [7]. The biofilm ECM-rich in insoluble EPS restricts the rinsing and buffering effect of saliva on surfaces while conferring protection to microorganisms from therapeutic agents by limiting their diffusion [8]. Thus, the absence of this type of ECM effect would minimize the capacity of acids to demineralize dental surfaces in the presence of saliva, being an attractive way to control the formation and accumulation of pathogenic biofilms and tooth decay [24].

Current approaches to control virulent biofilms in the mouth are very limited. Chlorhexidine is a broad-spectrum bactericidal agent that suppresses mutans streptococci levels in saliva but is less effective against biofilms (as revised by Mattos-Graner et al., [15]). Fluoride is the mainstay of caries prevention; however, it offers incomplete protection against caries and does not address the infectious aspects of the disease efficiently [25]. Fluoride is mainly involved in the remineralization process and slightly affects bacterial metabolism, reducing acid production [26]. Therefore, new anti-biofilm agents have been extensively searched.

When *S. mutans* was used as a single pathogen in vitro anti-plaque and in vivo anti-caries studies to test the effect of propolis (a natural, non-toxic beehive product), some compounds were identified as effective. Among them are worth highlighting a bioflavonoid (myricetin) and a terpenoid (*tt-*farnesol) [27–29]. Myricetin is an effective inhibitor of Gtfs enzymes in solution and reduces the expression of the *gtfBC* genes [30, 31], meanwhile *tt*-farnesol targets the cytoplasmatic membrane, decreasing acid tolerance of *S. mutans* [24, 28, 32]. Therefore, the combination of alternative agents and fluoride to improve the action that each one presented separately is an interesting strategy for anti-biofilm therapies [28, 31], even more when used against a more pathogenic setting (i.e., *S. mutans* and *C. albicans* dual-species biofilm [12]). Thus, this study evaluated the effect of *tt*-farnesol and myricetin on *S. mutans* and *C. albicans* dual-species biofilm, especially on exopolysaccharides found in the ECM.

## Methods

### Test agents

The test agents were *tt*-farnesol, myricetin, chlorhexidine digluconate, and sodium fluoride, which were obtained from Sigma-Aldrich Co (Catalog numbers: 277,541, 70,050, C9394, and 71,519, respectively). This study evaluated seven distinct treatment groups: vehicle (V - 15% ethanol solution into 2.5 mM phosphate buffer, pH 6.0, used to dissolve the agents just prior treating biofilms); Myricetin – 2 mM (M); Farnesol – 4 mM (F); Myricetin+Farnesol (MF); Sodium fluoride – 250 ppm (250); Myricetin + Farnesol + Fluoride (MF250), and chlorhexidine digluconate solution 0.12% (CHX; Sigma); these concentrations were chosen based on data from previous studies [28, 30–33].

### Biofilm formation on saliva-coated hydroxyapatite discs and topical treatments

#### Experimental design

**Fig. 1:**
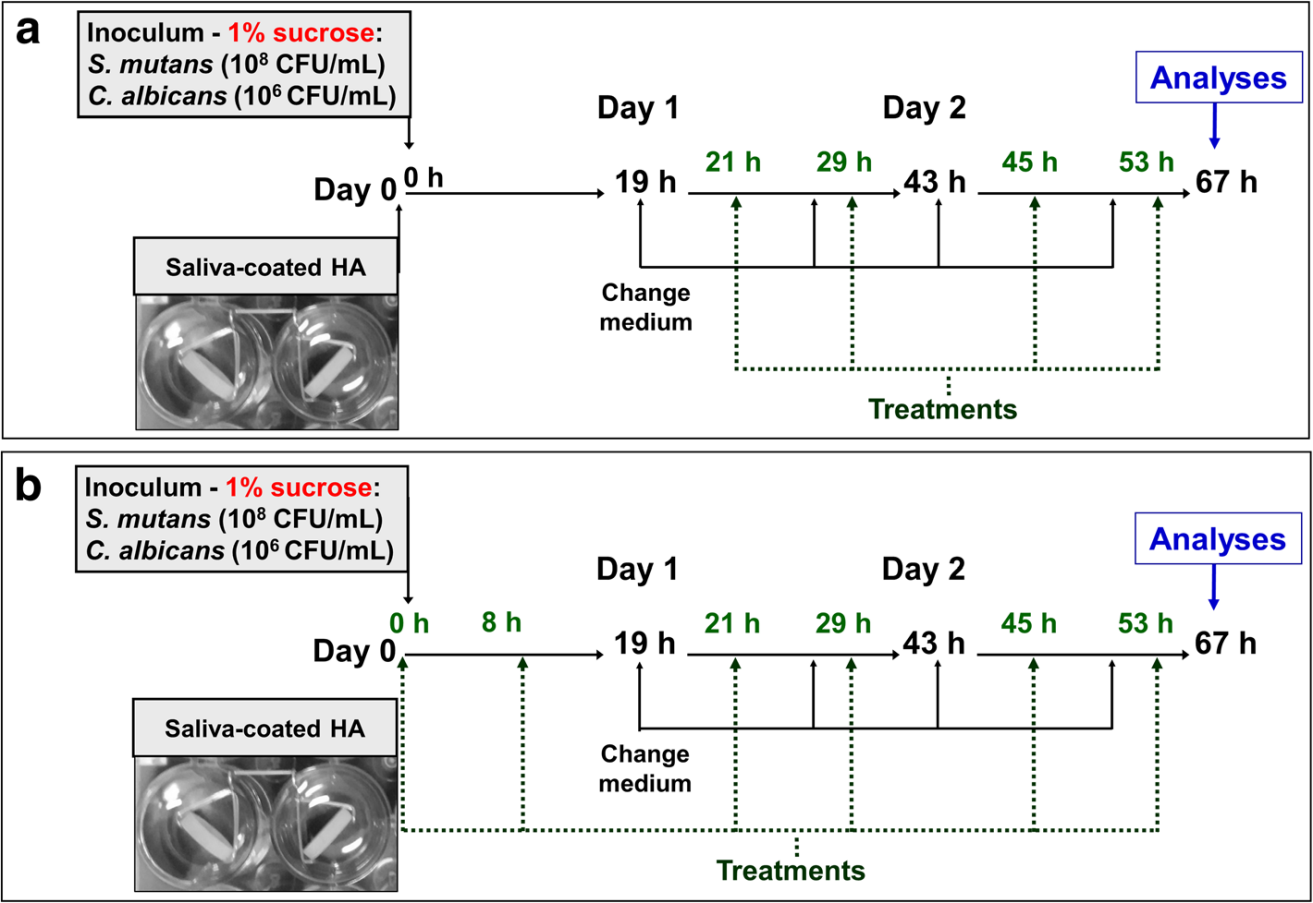
Treatment regimens used for topical application. **a** Depicts the regimen one used to accesses the effect on pre-formed biofilms (treatments application starts at 19 h of biofilm development). **b** Depicts the regimen two used to evaluate the effect on biofilm assembly and accumulation (treatments application on saliva-coated hydroxyapatite discs before microbial inoculation)

Two therapeutics regimens were performed (Fig. 1). In the first regimen, biofilms were topically treated after 21 h of development to evaluate the effect of agents on pre-formed biofilms (regimen one). In the second therapeutic regimen, the treatments were applied before microbial inoculation (i.e., the saliva-pellicle surface was treated) and after 8 h of biofilm growth to evaluate the preventive effect on biofilm assembly and accumulation (regimen two). The times of culture medium exchange and additional treatments were the same for both regimens until 67 h of each experiment.

#### Saliva-pellicle formation

The hydroxyapatite discs (surface area of 2.7 ± 0.2 cm^2^, Clarkson Chromatography Products Inc., PA, USA) were placed vertically into custom made holders (two discs per holder) and sterilized by autoclaving. To generate saliva-coated hydroxyapatite (sHA), discs were hydrated during 20 min with sterilized MiliQ water transferred to 24-well plates containing human stimulated whole saliva filtered sterilized (0.22 μm low protein binding polyethersulfone membrane filter), and incubated (37 °C, 75 RPM, 1 h) [34]. Saliva collection was approved by the Institutional Ethical Committee (CAAE: 31,725,114.8.0000.5416). Each apparatus with discs was removed from saliva, dip-washed twice into adsorption buffer (AB buffer: 50 mM KCl, 1 mM KPO_4_, 1 mM CaCl_2_, 1 mM MgCl_2_, in dd-H_2_O, pH 6.5), being ready to for biofilm formation and/or treatment, depending on the treatment regimen.

#### Biofilm preparation

The strains *S. mutans* UA159 and *C. albicans* SC5314 were used to prepare dual-species biofilms. The strains were grown on blood agar plates (48 h / 37 °C / 5% CO_2_). Five to ten colonies of each microorganism were inoculated into 10 ml of culture medium: tryptone with yeast extract containing 1% of glucose (TYE+ 1% glucose) and incubated (37 °C / 5% CO_2_). After 16 h, 1:20 dilutions of each starter culture were performed in TYE+ 1% glucose medium, and the cultures were grown until mid-log growth phase (OD_600nm_ 0.71 ± 0.27 for *S. mutans,* and 0.97 ± 0.03 for *C. albicans*). A dual-species inoculum was prepared with a defined population (*S. mutans* 2 × 10^8^ CFU/mL and *C. albicans* 2 × 10^6^ CFU/mL; CFU: colony forming units), in TYE with 1% sucrose [12]. In regimen one, sHA discs were placed into this inoculum, and discs were left undisturbed and incubated (37 °C/ 5% CO_2_) to allow initial biofilm formation. In regimen two, sHA discs were topically treated, then incubated into biofilm inoculum (37 °C/ 5% CO_2_), and after 8 h these biofilms were treated again. After 19 h of incubation for both regimens, the culture medium was replaced. This procedure was repeated twice daily (at 8 a.m. and 4 p.m.) and the pH of the spent medium was measured (Fig. 1). The biofilms were treated twice-daily 2 h after culture medium change (at 10 a.m. and 6 p.m.) until the end of the experiment (67 h-old biofilms).

#### Treatments

The biofilms were exposed to the test agents four times in the regimen one and six times in the regimen two, by topically dripping 160–240 μL of each treatment during 1.5 min. Rinses of sHA discs/biofilms into saline solution (0.89% NaCl) were performed before and after treatments to remove any excess of culture medium or treatment. After treatments, discs were placed back into the culture medium in the 24-well plates. Biofilms assays were performed in duplicate in at least four different experiments (only CHX were done in quadruplicate in two experiments because there was less biofilm formation).

#### Biofilm analyses

At the end of the experimental period (67 h), biofilms were dip-washed in saline solution. Each biofilm (disc) was transferred to a glass tube containing 1 mL of saline solution. Next, 1 mL of saline solution was used to wash the walls of each tube. The glass tubes with biofilms/discs were placed inside a Becker containing distilled water and subjected to water bath sonication (Kondortech Digital Ultrasonic Cleaner, Kondortech Indústria e Comércio Ltda., São Carlos, Brazil) during 10 min. A sterilized metal spatula was used to scrape off any remaining biofilm from each disc surface and the 2 mL of each biofilm suspension were transferred to a new 15 mL tube. Next, each glass tube was washed with 3 mL of saline solution, which were transferred to the tube containing the initial 2 mL, yielding a 5-mL total biofilm suspension per biofilm/disc. The 5 mL of each biofilm suspension were sonicated using a probe at 7 W during 30 s (Q125, Q Sonica). An aliquot of each suspension was used for a 10-fold serial dilution to determine the number of CFU by plating onto blood agar plates (37 °C/ 5%CO_2_/ 48 h).

The remaining suspension volume of each biofilm was centrifuged (3942 rfc/20 min/4 °C), the supernatant was saved (supernatant 1) and each pellet was washed twice with sterile water generating supernatants 2 and 3. Three supernatants (totaling 10 mL) were used to quantify water soluble polysaccharides (WSP). Each biofilm pellet was suspended in 2.6 mL of MiliQ water. Each biofilm suspension was used to analyze insoluble dry weight (biomass) and alkali soluble or water-insoluble polysaccharides (ASP). The biofilm aliquot for ASP extraction was dried (Speed Vac Concentrator RVC 2–18 CD Plus, Christ). The resulting pellet was weighted and used to extract ASP using 1 N NaOH (0.3 mL of 1 N NaOH per 1 mg of biofilm dry weight). The quantification of WSP and ASP was performed using phenol-sulfuric acid colorimetric assay with glucose as standard [35].

### Statistical analyses

The statistical analyses were performed using Prism7 GraphPad software (GraphPad Software, Inc., La Jolla, CA, USA), employing a significance level fixed at 5%. The data were analyzed by Shapiro-Wilk normality test. The data with normal distribution were subjected to parametric tests one-way ANOVA, followed by Tukey’s multiple comparisons test (WSP and ASP for regimen one; *S. mutans* and *C. albicans* population, and WSP for regimen two); while data without normal distribution were evaluated by non-parametric Kruskal-Wallis test, followed by Dunn’s multiple comparisons test (biomass and *S. mutans* and *C. albicans* population for regimen one; biomass and ASP for regimen two). Moreover, the pH results were analyzed by two-way ANOVA, considering the factors treatment and time, followed by Tukey’s multiple comparisons test.

## Results

### pH of spent medium

**Fig. 2:**
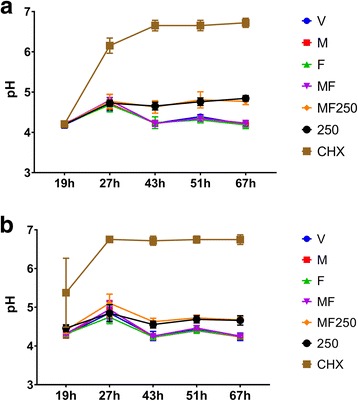
pH of spent media of treated biofilms. **a** Represents the data generated for regimen one, while (**b**) for regimen two. The pH values were measured when the culture media was changed. V: vehicle; F: *tt-*farnesol; M: myricetin; MF: *tt*-farnesol + myricetin; MF250: fluoride + *tt-*farnesol + myricetin; 250: fluoride (250 ppm); CHX: chlorhexidine. The data shown are averages, and error bars indicate the standard deviation. Statistical analysis is not depicted in the graphs

The discs were placed in a medium with a neutral pH, and during the first 19 h, the metabolism of carbohydrates by microorganisms acidified the milieu, generating a pH drop independently of the treatment regimen (Fig. 2). At 19 h, for treatment regimen two, microorganisms under treatment with positive control CHX caused less acidification. In addition, three types of behaviors were observed depending on different agents used to treat the biofilms (in both regimens). Treatments that had fluoride (MF250 and 250) revealed slightly higher pH values when compared to the groups without fluoride (V, F, M, MF) at 43 h, 51 h and 67 h (*p* < 0.05). Only biofilms treated with the positive control CHX presented pH higher than 6.5 for both regimens.

### Biomass of biofilms after treatments

**Fig. 3:**
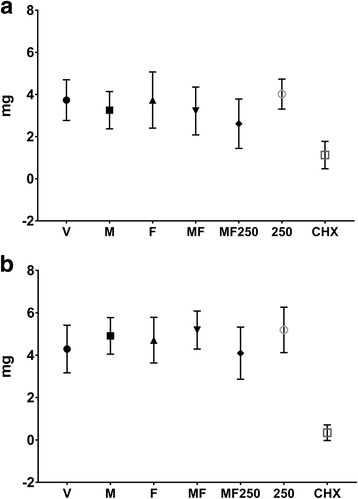
Biomass of treated biofilms. **a** represents the data generated for regimen one, while (**b**) for regimen two. V: vehicle; F: *tt-*farnesol; M: myricetin; MF: *tt*-farnesol + myricetin; MF250: fluoride + *tt-*farnesol + myricetin; 250: fluoride (250 ppm); CHX: chlorhexidine. The data shown are averages, and error bars indicate the standard deviation

For regimen one, the treatments V, M, F, MF and 250 lead to similar biomass accumulation (*p* > 0.05; Fig. 3a), and these biomasses values were higher than the biomass of the positive control CHX-treated biofilms (*p* < 0.05); while the biomass of biofilms treated with MF250 was not statistically different from of CHX-treated biofilms (*p* > 0.05). For regimen two, the biomasses of all treatments were similar (*p* > 0.05) and higher than that of CHX-treated biofilms (*p* < 0.05; Fig. 3b). The biomasses of biofilms submitted to regimen two were slightly higher than those biofilms submitted to regimen one, except for CHX-treated biofilm. Biomass of CHX-treated biofilms with regimen two was lower than that of biofilms subjected to regimen one. Thus, when CHX was applied directly to salivary-pellicle, this agent hinders more effectively biofilm accumulation.

### Viable microbial population recovered after treatments

**Fig. 4:**
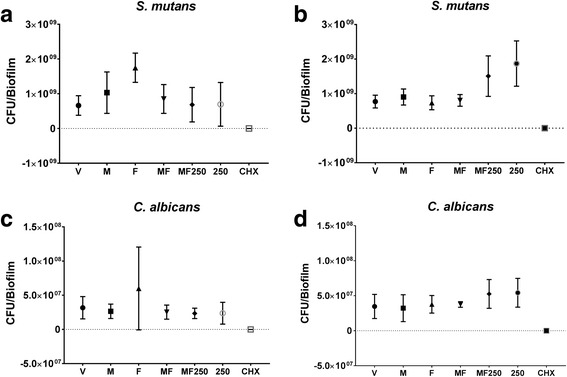
Microbial population of *S. mutans* and *C. albicans* after treatment of biofilms. Data are presented as colony forming unit (CFU) per biofilm. **a** and **c** represent data generated for regimen one, while (**b**) and (**d**) correspond to regimen two. V: vehicle; F: *tt-*farnesol; M: myricetin; MF: *tt*-farnesol + myricetin; MF250: fluoride + *tt-*farnesol + myricetin; 250: fluoride (250 ppm); CHX: chlorhexidine. The data shown are averages, and error bars indicate the standard deviation

The microbial population of *S. mutans* and *C. albicans* recovered from treated biofilms are shown in Fig. 4. In both regimens evaluated, only the positive control CHX showed a major reduction in both species (vs. all other treatments, *p* < 0.05). For regimen one, biofilms treated with M, MF, MF250, and Fluoride presented similar *S. mutans* counts to biofilms treated with the negative control (vehicle; *p* > 0.05). Moreover, it was observed a larger number of CFU for both species when biofilms were treated with only *tt-*farnesol (F) in regimen one. For regimen two, *S. mutans* population was higher for biofilms subjected to treatments containing fluoride (MF250 and 250), and again, CHX-treated biofilms presented lower quantities of both *S. mutans* and *C. albicans* (*p* < 0.05).

### Exopolysaccharides present in the extracellular matrix

**Fig. 5:**
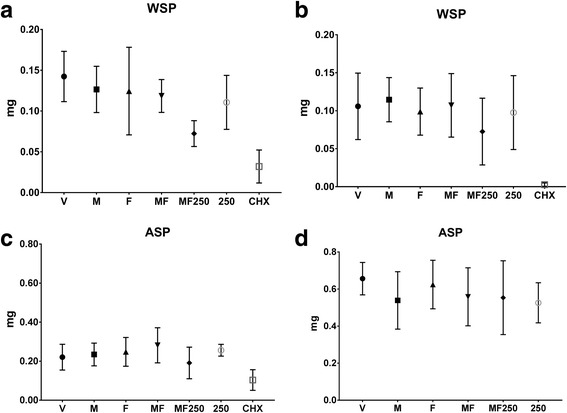
Exopolysaccharides quantities in the ECM after treatments. Both water soluble (WSP) and water insoluble (ASP) exopolysaccharides in the extracellular matrix are represented in milligrams (mg). (**a**) and (**c**) represent data generated for regimen one, while (**b**) and (**d**) correspond to regimen two. Panels **a** and **b** depict WSP data and panels **c** and **d** show ASP data. V: vehicle; F: *tt-*farnesol; M: myricetin; MF: *tt*-farnesol + myricetin; MF250: fluoride + *tt-*farnesol + myricetin; 250: fluoride (250 ppm); CHX: chlorhexidine. The data shown are averages, and error bars indicate the standard deviation

The quantification of exopolysaccharides present in the extracellular matrix is depicted in Fig. 5. For regimen one, a lower quantity of water soluble polysaccharides (WSP) was observed in the matrix of biofilms treated with CHX compared to all treatments (*p* ≤ 0.0001); except for MF250, which features similar amount (*p* = 0.1514). Moreover, WSP amount was lower in the ECM of biofilms treated with MF250 (vs. F and M; *p* < 0.03), using the regimen one. Therefore, MF250 treatment in regimen one hindered WSP accumulation. Regarding water insoluble exopolysaccharides (ASP) in the ECM of biofilms subjected to regimen one, a lower amount was detected in biofilms treated with CHX (vs. all treatments – *p* < 0.05; except for MF250 and vehicle). For regimen two, all treatments did not stand out against the negative control for both WSP and ASP, showing that this regimen may not be effective to hinder EPS production (Fig. 5).

## Discussion

Due to the widespread of caries, costs and inconvenience that this disease generates [1, 2] exist a continuous interest in developing novel strategies to control cariogenic biofilms that act against its virulence traits. Trying to reproduce the complexity of oral biofilm is difficult because of the interference of many factors (e.g., complex microbiota, salivary flow, diet, others). The *S. mutans* and *C. albicans* dual-species biofilm model used here was to represent a critical clinical situation, since these two species are commonly found in the oral cavity and are proven pathogens when the host and environmental conditions enable them [14, 20–22]. Specifically, these two microorganisms were used to grow a biofilm characterized by its glucan-rich matrix [12]), typically found in cases of destructive caries, especially in S-ECC (as revised by Hajishengallis et al. [36]).

The use of chlorhexidine (a broad-spectrum antimicrobial agent) is considered the gold-standard therapy to control oral biofilms. However, chlorhexidine suppresses the oral microbiota [15] and has a restricted use (only for a period of 14 days), because of its collateral effects [37]. In addition, fluoride is the gold-standard for caries prevention, but it offers incomplete protection against the disease and inadequately treats the infectious aspect of caries [25]. Consequently, natural agents that could increase the effectiveness of topically applied fluoride have been investigated to improve oral care [24].

Here, trying to simulate human exposure to oral care products, treatments were applied twice daily during a brief time (1.5 min). It was observed a similar diminution of water-soluble EPS in the ECM when a combination therapy (MF250) and a positive control (0.12% CHX) were used. Therefore, MF250 affected negatively biofilm development, making these biofilms potentially less pathogenic. This effect in reducing exopolysaccharides is significant because the ability of microorganisms to synthesize glucan may be more important for virulence than its population itself [38], as a debilitated ECM may not provide an adequate 3D structure and stability for microorganisms in the biofilm. Moreover, saliva could perform its buffer activity, possibly decreasing the formation of acidic niches within dental biofilms.

However, even showing impact in the EMC composition, it was observed a pH drop in the culture media (except for CHX), reaching critical values that can trigger a demineralization process (5.5 for enamel and 6.5 for dentin) [39]. Nevertheless, there was a lower drop in pH when fluoride was present in the topical treatments, because it reduces the production of acids by microorganisms in biofilms and releases ions to assist in the remineralization process, which are the major pathways to prevent early caries lesions [26].

Although ECM in biofilms protects microorganisms and EPS negatively charged affects penetration (and antimicrobial activity) of CHX [8, 40], the inhibitory power of the positive control CHX was evident regardless of the treatment regimen employed. This effect could be explained because the CHX was designed to modify the integrity of cell membranes, causing dispersion of microbial components of low molecular weight, particularly in the surfaces of Gram-positive bacteria [41]. Moreover, when microbial cells are dead, there are fewer surfaces available for Gtfs adhesion and EPS synthesis. However, in cases of caries with rapid progression (i.e., S-ECC), chlorhexidine antifungal effectiveness must be confirmed on in vivo and in clinical studies, because there may be a different behavior in biofilms formed on teeth of live hosts where several factors may be interacting.

*C. albicans* has several virulence factors, including its capacity to switch its morphology that is influenced by quorum-sensing molecules (i.e.*,* tyrosol from yeast to hypha, and farnesol from hypha to yeast), biofilm formation, and control of nutrient competition [42–44]. Therefore, the use of *tt-*farnesol by itself may explain the higher numbers of *C. albicans*, compared to all other treatments in the regimen to disrupt pre-formed biofilms (regimen one), because farnesol stimulates yeast morphology. However, this data was opposite to a previous study that observed lower *C. albicans* population in single-species biofilms in the presence of farnesol [43]. Therefore, *C. albicans* in a dual-species biofilm with *S. mutans* may behave differently when challenged by therapeutic agents, which may benefit both species to maintain a symbiotic relationship, and this behavior may be dependent upon the treatment regimen employed.

Furthermore, when *tt-*farnesol was mixed with myricetin (MF) and with fluoride (MF250), the population of both species was higher than the ones after treatments with the other agents in the regimen that treated salivary pellicle, before microbial inoculation (regimen two), which was unexpected. Our theory was that topically treating the pellicle would prevent biofilm accumulation and microbial population increase (compared to the negative control vehicle). The reason for the distinct population outcome from both regimens is unclear because the quantity of ECM components was lower for treatments with combination therapy. Nevertheless, we hypothesize that the presence of residual treatment on the pellicle (in regimen two) could have elicited a stress response from the microorganisms to build up biofilm, leading to the higher biomass and ASP observed for this regimen, compared to the regimen one (treatment starting after 19 h of biofilm development). Additional studies are warranted to clarify this theory further.

In addition, change in drug concentration or increase in exposure time may improve the results in reducing the number of viable cells and EPS in the matrix. The treatment potential of MF250 was confirmed by reduction of matrix extracellular formation (WSP), similarly to the positive control (CHX) in regimen one. Therefore, additional research should be performed with molecules from natural sources, such as the ones used here, so that more positive results are collected, showing their pharmacological potential in the protection of oral health.

## Conclusions

The control of biofilm development using natural drugs showed favorable results regarding the formation of extracellular matrix. However, it may be necessary to change the concentration of the tested agents so that a more robust outcome is achieved, decreasing additional virulence characteristics of the biofilm.

## Abbreviations

3D: Tridimensional
ASP: Alkali soluble polysaccharides
CHX: Chlorhexidine digluconate
ECM: Extracellular matrix
EPS: Exopolysaccharides
F: Farnesol
M: Myricetin
MF: Myricetin + Farnesol;
MF250: Myricetin + farnesol + sodium fluoride
ppm: Parts-per-million
S-ECC: Severe early childhood caries
TYE: Tryptone yeast extract
V: Vehicle
WSP: Water soluble polysaccharides
